# Lord Denbigh's Idea

**Published:** 1921-03-26

**Authors:** 


### Lord Denbigh's Idea.

### A CRITICISM AND A PRACTICAL PROPOSAL.

The Earl of Denbigh, Chairman of the National Orthopædic Hospital, contributes some interesting suggestions to the *Review of Reviews* (March-April) in regard to hospital finance. After affirming the importance of maintaining the voluntary system, the chief arguments in its favour being clearly stated, Lord Denbigh expresses the opinion that too many committees are perhaps dealing with the present problem. "There could," he adds, "be probably constituted no more competent or independent body to advise upon the financial difficulties of London hospitals to-day than King Edward's Hospital Fund." It has, he notes, the confidence of the public and that of the hospitals; it is acquainted with the details of management or finance of almost every hospital in London; its detached and paternal interest has helped efficiency; its supervision is as little irksome as any can be.

Lord Denbigh then urges that the British Hospitals Association should not, through its London Regional Committee, supply and duplicate the information which King Edward's Fund already can give to Lord Cave's Committee. In regard to patients' payments, he records that at his own institution a system of payment has been in operation for many years, and so sympathetically enforced that the patients gladly co-operate. He criticises the proposal for factories and stores to "adopt" particular hospitals, because he thinks that it would lead to the "over-development" of one hospital at the expense of others, unless the contributions from the adopting businesses paid their sums into a central fund, which in London, preferably, he says, should be King Edward's.

He also thinks that if the education authority should pay for all school children treated in hospital, it would "increase rates and probably taxes," and tend to introduce, if not State control, at all events the "burdensome paraphernalia of bureaucracy." Government Department returns multiply themselves, and further payments by public authorities "lessen responsibility," and "lead to difficulties of treatment. Most hospitals have experienced the difficulties which arise in this way when dealing with patients sent by some Medical Referees under the Ministry of Pensions, and some education medical officers." He therefore urges hospitals "to be very chary of encouraging payment on any large or general scale by public authorities for cases sent by the latter, even in respect of those patients who are by statute chargeable to public funds."

Lord Denbigh advocates paying wards, provided the profit be kept small: "In one case in London the fees charged being no less prohibitive than those of the nursing homes themselves!" The difficulties in the way of relieving voluntary contributions from income-tax and death-duties seem to him inseparable; but he finds much less difficulty in exempting "recognised voluntary hospitals from the payment of rates," and favours this plan. For the moment he suggests that, to ensure the permanence of the voluntary system, the "Government should become a temporary annual subscriber to King Edward's Hospital Fund, its subscriptions to be allotted for general maintenance, and the amount and conditions to be upon "an agreed basis." This plan, he points out, would incur no extra administrative expenses, and create no new Government Department, while the money would go direct to its proper object.

